# Quinolone nonsusceptibility among enteric pathogens isolated from international travelers – Foodborne Diseases Active Surveillance Network (FoodNet) and National Antimicrobial Monitoring System (NARMS), 10 United States sites, 2004 – 2014

**DOI:** 10.1371/journal.pone.0225800

**Published:** 2019-12-04

**Authors:** Julian E. Grass, Sunkyung Kim, Jennifer Y. Huang, Stephanie M. Morrison, Andre E. McCullough, Christy Bennett, Cindy R. Friedman, Anna Bowen

**Affiliations:** 1 Division of Foodborne, Waterborne, and Environmental Diseases, Centers for Disease Control and Prevention, Atlanta, Georgia, United States of America; 2 Division of Global Migration and Quarantine, Centers for Disease Control and Prevention, Atlanta, Georgia, United States of America; School of Pathology, National Health Laboratory Service (NHLS) and University of the Witwatersrand, South Africa, SOUTH AFRICA

## Abstract

Gastrointestinal illnesses are the most frequently diagnosed conditions among returning U.S. travelers. Although most episodes of travelers’ diarrhea do not require antibiotic therapy, fluoroquinolones (a type of quinolone antibiotic) are recommended for treatment of moderate and severe travelers’ diarrhea as well as many other types of severe infection. To assess associations between quinolone susceptibility and international travel, we linked data about isolate susceptibility in NARMS to cases of enteric infections reported to FoodNet. We categorized isolates as quinolone-nonsusceptible (QNS) if they were resistant or had intermediate susceptibility to ≥1 quinolone. Among 1,726 travel-associated infections reported to FoodNet with antimicrobial susceptibility data in NARMS during 2004–2014, 56% of isolates were quinolone-nonsusceptible, of which most (904/960) were *Campylobacter*. International travel was associated with >10-fold increased odds of infection with quinolone-nonsusceptible bacteria. Most QNS infections were associated with travel to Latin America and the Caribbean (390/743; 52%); however, the greatest risk of QNS infection was associated with travel to Africa (120 per 1,000,000 passenger journeys). Preventing acquisition and onward transmission of antimicrobial-resistant enteric infections among travelers is critical.

## Introduction

Travelers make >1 billion international tourist arrivals worldwide annually [1]; gastrointestinal illnesses are the most frequently diagnosed conditions among returning U.S. travelers [2]. Although most episodes of travelers’ diarrhea do not require antibiotic therapy, fluoroquinolones (a type of quinolone antibiotic) are often dispensed for adult travelers and recommended for treatment of moderate and severe travelers’ diarrhea [3]. Resistance to quinolones has been linked to international travel [4, 5]. We describe quinolone susceptibility among isolates from travel-associated enteric infections reported in the United States during 2004–2014.

## Methods

FoodNet conducts active, population-based surveillance for enteric infections in 10 U.S. sites (Connecticut, Georgia, Maryland, Minnesota, New Mexico, Oregon, Tennessee, and selected counties in California, Colorado, and New York) [6]. The FoodNet surveillance area includes 15% of the United States population, or approximately 48 million people [6]. FoodNet collects data on international travel in the 7 days before illness onset [7] and hospitalization within 7 days of specimen collection. FoodNet retains a single report for cases in which multiple isolates are collected within a 30-day period.

NARMS at CDC monitors prevalence of and trends in antimicrobial resistance among enteric bacteria isolates from humans [8]. State and local public health laboratories systematically submit every 20^th^ nontyphoidal *Salmonella* (NTS), *Shigella*, and *Escherichia coli* O157 (O157) isolate to CDC’s NARMS laboratory for susceptibility testing; for *Campylobacter*, a site-specific percentage of isolates comprise a convenience sample [8]. NTS, O157, and *Shigella* isolates were tested using broth microdilution (Sensititre^®^, Trek Diagnostics, part of Thermo Fisher Scientific, Cleveland, OH) to determine the minimum inhibitory concentrations for ciprofloxacin and nalidixic acid (quinolones) [8]. Methods for susceptibility testing of *Campylobacter* and interpretive criteria are described in a 2018 CDC report [8]. Using interpretative criteria from the Clinical and Laboratory Standards Institute [9], we categorized isolates as quinolone nonsusceptible (QNS) if they were resistant or had intermediate susceptibility to ≥1 quinolone.

To assess associations between quinolone susceptibility and international travel, we linked FoodNet and NARMS data for *Campylobacter*, NTS, *Shigella*, and O157 isolates collected during 2004–2014 by laboratory identification number, specimen collection date, specimen source, patient age, county of residence, and sex. We categorized regions as defined by the World Health Organization [10]. We conducted multivariable logistic regression using SAS 9.3 (Cary, NC) with QNS as a binary outcome and travel as a primary independent variable, adjusting for year. We created one model for all pathogens combined, and another with an interaction term between travel and pathogen to assess the pathogen as an effect modifier.

We accounted for the NARMS sampling scheme by multiplying the number of overall or QNS travel-associated infections by the NARMS sampling rate (e.g., we multiplied the number of *Shigella* isolates by 20 because NARMS received every 20^th^ Shigella isolate). To estimate risk of enteric infection among travelers, we divided the number of travel-associated infections by estimates of international aviation passenger journeys terminating in FoodNet sites during 2010–2014 [6, 11]. Passenger journeys are not unique, individual travelers. We report infections per 1,000,000 passenger journeys.

## Results

**Fig 1:**
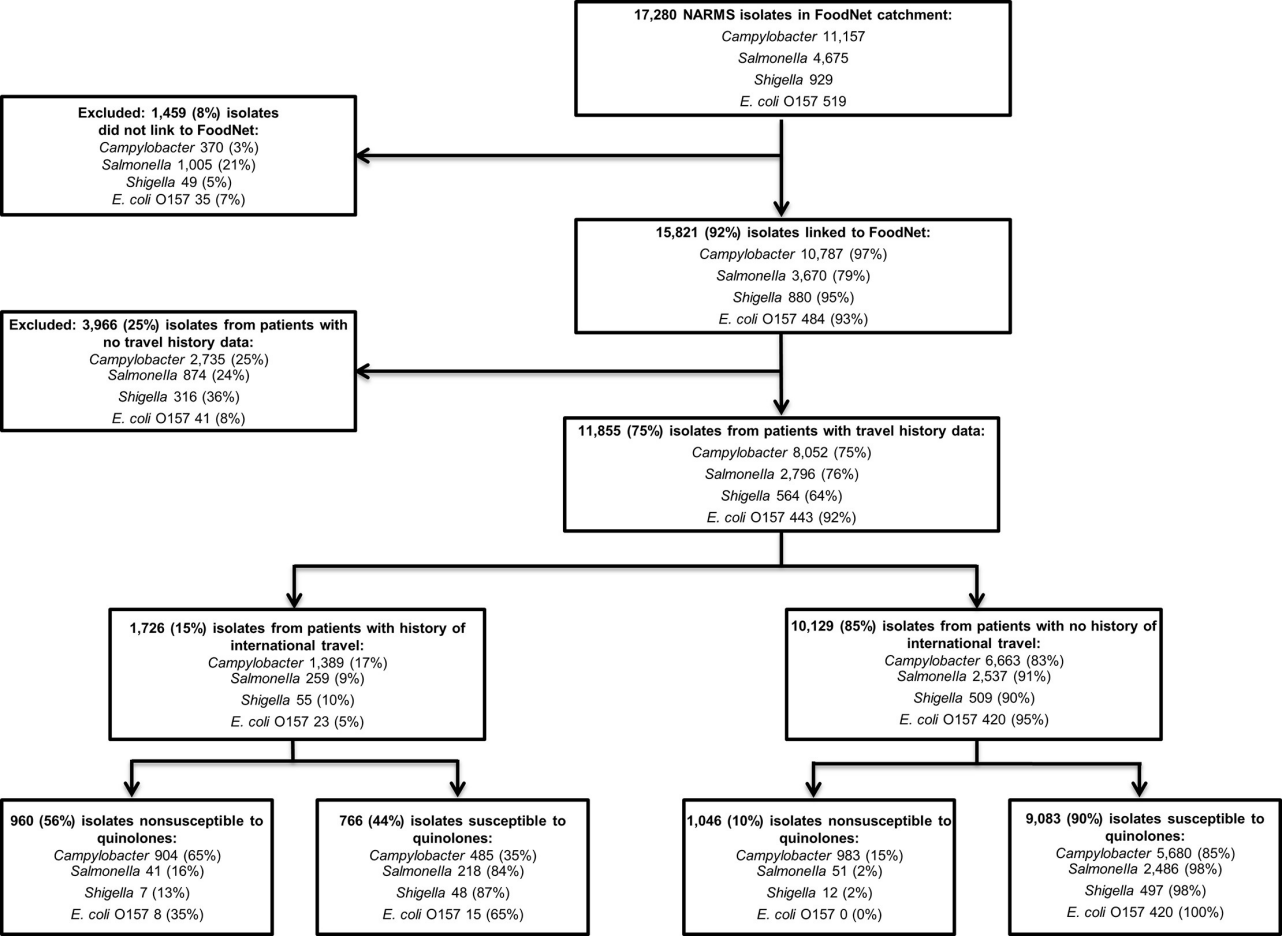
Number and percentage of National Antimicrobial Resistance Monitoring System (NARMS) isolates included in the study from patients in the FoodNet catchment area with a reported history of international travel, by pathogen, 2004–2014.

Overall, 92% (15,821/17,280) of NARMS isolates from FoodNet sites were linked to cases reported to FoodNet (Fig 1). The percentage linked varied by pathogen: *Campylobacter* (97%), *Shigella* (95%), O157 (93%), and NT*S* (79%). Travel data were available for 11,855 (75%) of the linked patients, including 8,052 (75%) infected with *Campylobacter*, 2,796 (76%) NTS, 564 (64%) *Shigella*, and 443 (92%) O157; overall, 1,726 (15%) reported travel (17% *Campylobacter*, 10% *Shigella*, 9% NTS, and 5% O157). Among travel-associated isolates, 960 (56%) were QNS; most (904) were *Campylobacter* (Fig 1). QNS and ciprofloxacin nonsusceptibility were most common among travel-associated *Campylobacter* isolates (65%, 65%, respectively), followed by O157 (35%, 0%), NTS (16%, 16%), and *Shigella* (13%, 7%).

We found a strong association between QNS and travel among all enteric pathogens combined (odds ratio (OR) [95% confidence interval (CI)] = 11.4 [10.2–12.8], p<0.001), and between QNS and each enteric pathogen individually (Table 1). The odds of QNS among enteric isolates increased annually (OR [95% CI] = 1.1 [1.0–1.1], p<0.001).

**Table 1:** Distribution and adjusted odds ratios of quinolone-nonsusceptible infections for international travelers compared with non-travelers, by pathogen, Foodborne Diseases Active Surveillance Network and National Antimicrobial Resistance Monitoring System, United States, 2004–2014.

	Travel-associated					Non-Travel-associated							
	Total (No.)	CIP^‡^ (No.)	NAL^‡^ (No.)	Total QNS^§^ (No.)	% QNS^§^	Total (No.)	CIP^‡^ (No.)	NAL^‡^ (No.)	Total QNS^§^ (No.)	% QNS^§^	OR*	(95% CI)	P-value
All pathogens combined	1726	943	951	960	55.6	10129	1011	1024	1046	10.3	11.4	(10.2–12.8)	<0.001
By pathogen													
*Campylobacter*	1389	898	903	904	65.1	6663	963	976	983	14.8	11.1	(9.8–12.7)	<0.001
Nontyphoidal *Salmonella*	259	41	33	41	15.8	2537	45	36	51	2.0	9.3	(6.0–14.4)	<0.001
*Shigella*	55	4	7	7	12.7	509	3	12	12	2.4	6.2	(2.3–16.6)	<0.001
*E*. *coli* O157^†^	23	0	8	8	34.8	420	0	0	0	0.0	> 1000	(0 –>1000)	Undetermined

* Odds ratio; adjusted for calendar year to account for yearly variation.

†Odds ratio and 95% confidence interval for *E*. *coli* O157 cases were estimated to be >1000 because all quinolone-nonsusceptible infections occurred in international travelers (quasi-complete separation)

‡CIP: nonsusceptible to ciprofloxacin; NAL: nonsusceptible to nalidixic acid

§QNS: quinolone nonsusceptible. All isolates were tested for susceptibility to ciprofloxacin and nalidixic acid; some isolates were nonsusceptible to only one of these two antimicrobials.

Travelers were more likely to be hospitalized than non-travelers (OR [95% CI] = 3.2 [2.6–3.8], p<0.001); however, odds did not differ by quinolone susceptibility. Compared with *Campylobacter*, hospitalization was more common among patients with O157 (OR [95% CI] = 4.3 [3.5–5.2], p<0.001) and NTS (OR [95% CI] = 1.9 [1.7–2.2], p<0.001) infections.

**Fig 2:**
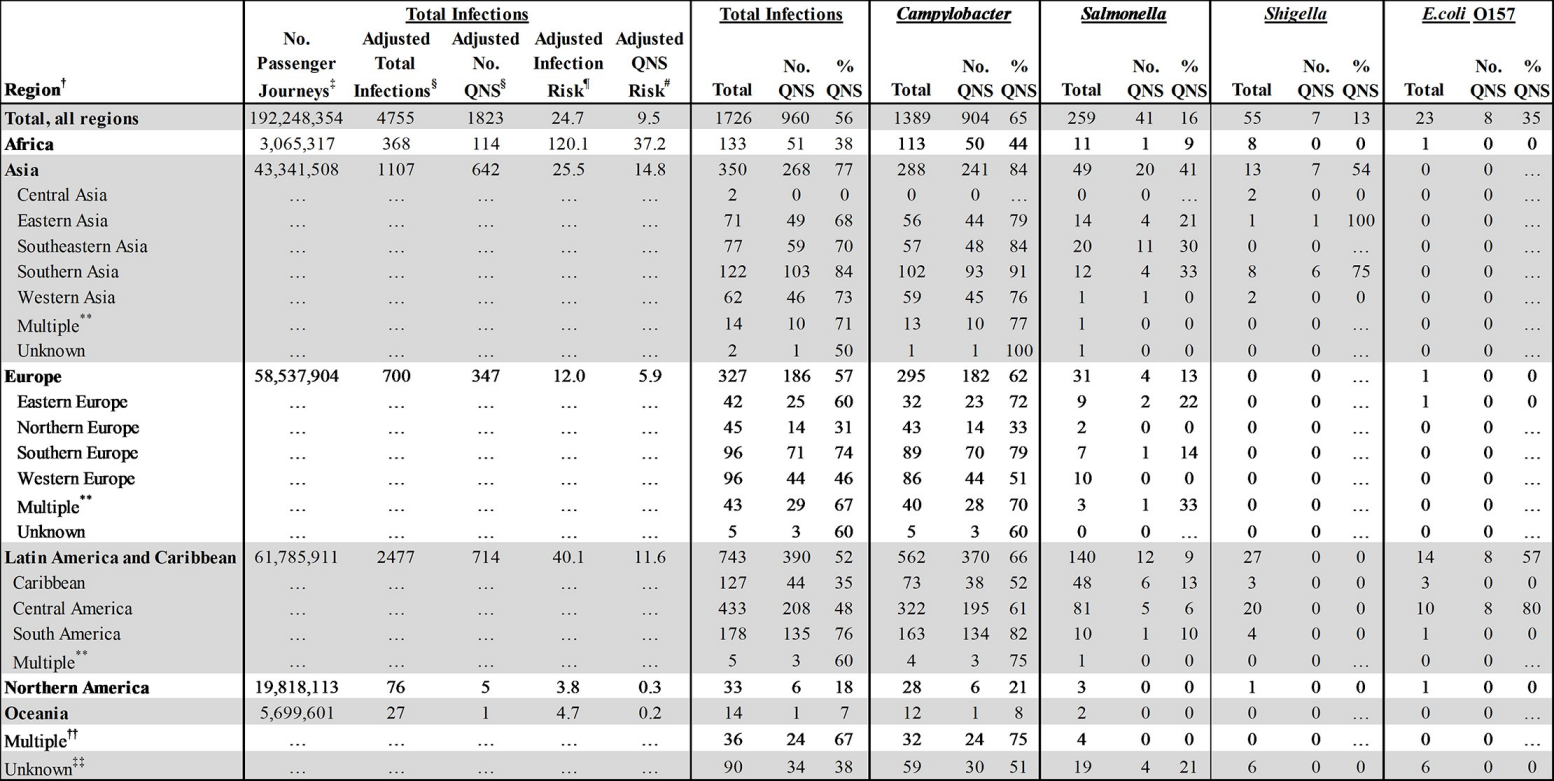
Number, percentage, and risk (per 1,000,000 air passenger journeys) of quinolone-nonsusceptible (QNS) infections in US travelers, by pathogen and region—Foodborne Diseases Active Surveillance Network and National Antimicrobial Resistance Monitoring System, United States, 2004–2014*.*. Risk estimates were calculated for 2010–2014 because data about numbers of travelers to each region were not available for 2004–2009. †World Health Organization regions (*9*) ‡The estimated number of aviation passenger journeys on direct or multi-leg international flights terminating in the FoodNet catchment area during 2010–2014, as reported by the International Air Transport Association (*10*). Passenger journeys are not unique, individual travelers. Overland travelers were not included. §Number of travel-associated, and quinolone-nonsusceptible enteric cases during 2010–2014 adjusted to account for the NARMS sampling scheme by using a series of pathogen-specific multipliers. NARMS collects every 20^th^ *NTS*, *Shigella*, and O157 isolate, so we multiplied the number of cases with these infections by 20. For *Campylobacter*, the multiplier varied, according to the proportion of isolates submitted by site: we applied no multiplier if all isolates were submitted (Connecticut, Oregon, and Tennessee); multiplied by 2 if every 2^nd^ isolate is submitted (California, Colorado, Georgia, Maryland, and New York); multiplied by 3 if every 3^rd^ isolate is submitted (New Mexico); and multiplied by 5 if every 5^th^ isolate is submitted (Minnesota). ¶Adjusted risk of diagnosis with an enteric infection after return to the United States per 1,000,000 passenger journeys.#Adjusted risk of diagnosis with a quinolone-nonsusceptible infection after return to the United States per 1,000,000 passenger journeys. **Case traveled to more than one sub-region in the same region. ††Case traveled to more than one region. ‡‡Travel destination was not reported.

Destination was reported by 1,636/1,726 (95%) travelers (Fig 2). The most common destinations were Latin America and the Caribbean (LAC) (45%), Asia (21%), and Europe (20%). QNS was most common among isolates from travelers to Asia (268, 77%); among these, QNS was detected in 241 (84%) *Campylobacter*, 20 (41%) NTS, and 7 (54%) *Shigella* isolates. Travelers to LAC accounted for the greatest number of QNS infections (390, 52%), including 370 (66%) *Campylobacter* and all 8 (57%) of the QNS O157 infections. Although fewer infections were associated with travel to Europe, more than half such infections were QNS (186, 57%). QNS was least common among isolates from travelers to Africa (51, 38%), Northern America (NA) exclusive of the United States (6, 18%), and Oceania (1, 7%). The largest numbers of QNS pathogens were isolated from travelers returning from Mexico (n = 152), India (n = 75) and Peru (n = 59).

**Fig 3:**
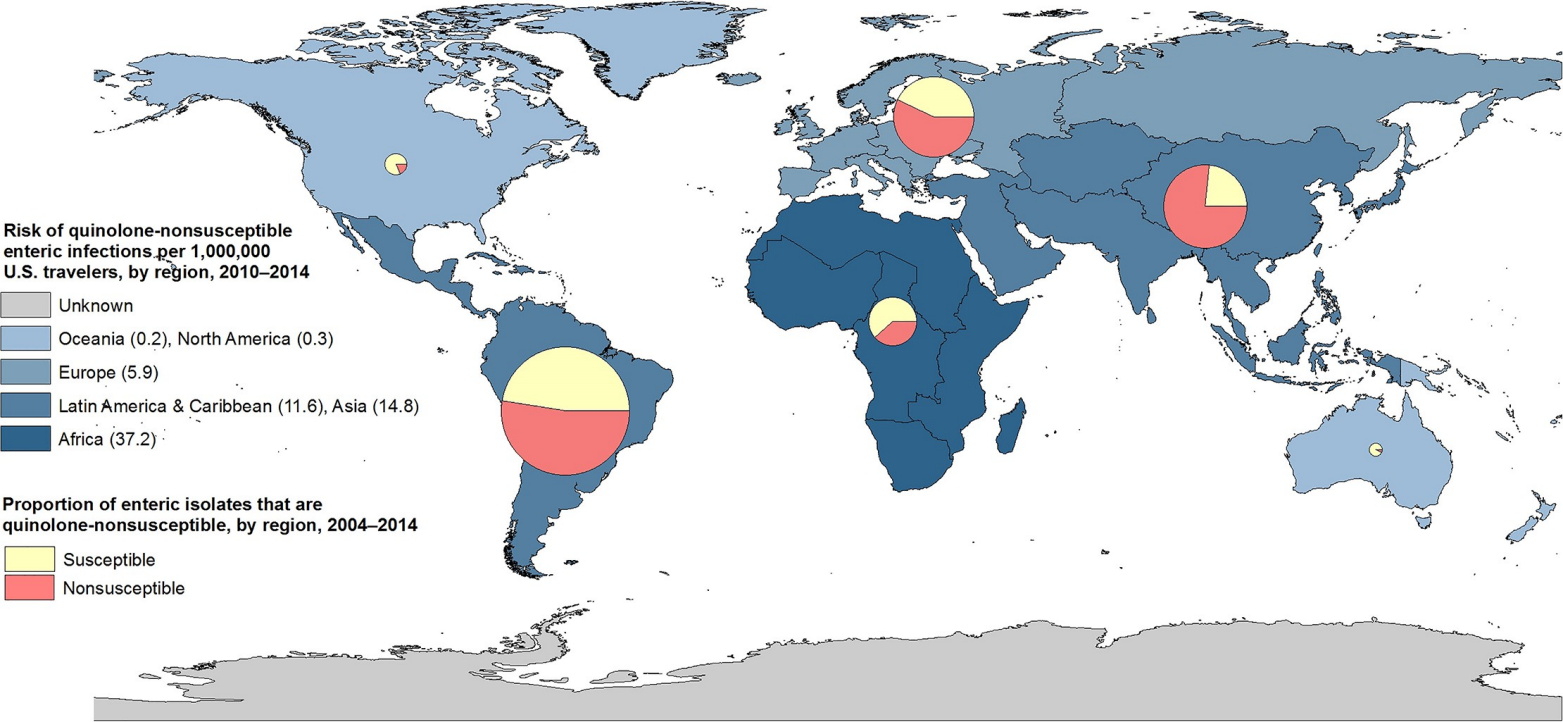
Proportion and risk of quinolone-nonsusceptible enteric infections in US travelers, by region, 2004–2014*. *Displays, by region, both the risk of quinolone-nonsusceptible enteric infection (differentiated by map shading) and the proportion of enteric isolates that are quinolone-nonsusceptible (presented as pie chart). Size of pie is proportional to the number of isolates tested per region.

Risk of acquiring an enteric infection while abroad varied by region. Risk for any enteric infection was 25 per 1,000,000 passenger journeys. Travel to Africa was associated with the greatest risk (120), followed by LAC (40), Asia (26), Europe (12), Oceania (5), and NA (4). Risk of acquiring a QNS infection also varied by travel destination (Figs 2 and 3). The estimated overall risk was 10 QNS infections per 1,000,000 passenger journeys; travel to Africa was associated with the highest risk (37), followed by Asia (15), LAC (12), Europe (6), NA (0.3), and Oceania (0.2). We were unable to assess risk of QNS infection by country because we lacked information about numbers of US travelers to each country.

## Conclusions

Using population-based, active surveillance data, we found that among persons with enteric bacterial infections in the United States, international travel is associated with more than ten-fold increased odds of QNS. Among 1,726 travel-associated isolates in our study, 56% were QNS and 55% were ciprofloxacin-nonsusceptible. QNS was most common among *Campylobacter* isolates, followed by O157, NTS, and *Shigella*. Patients infected with drug-resistant pathogens may experience more severe illness, hospitalizations, and deaths than those infected with drug-susceptible pathogens [12, 13]; the high proportions of travel-associated isolates with QNS suggest that additional efforts to prevent and detect such infections among international travelers are needed. Additionally, infected travelers may transmit drug-resistant pathogens or genes conferring drug resistance during travel and after returning home [4, 14, 15, 16]. Quinolones are used to treat many serious infections and account for ~20 million U.S. prescriptions annually; preserving their utility is critical [17].

The burden and risk of QNS infections varied by travel destination. Most QNS infections were associated with travel to LAC, likely due to the high volume of travel to this region; travel to Mexico was associated with 16% of all QNS infections. The proportion of enteric infections with QNS was highest among travelers to Asia (77%). However, the greatest risk of QNS infection was associated with travel to Africa, where risk was over twice that for LAC or Asia and five-fold compared with Europe, perhaps because, as shown in previous studies, travelers to Africa have the greatest risk of acquiring enteric infections [7, 18].

This study had limitations. Diarrhea is typically self-limited and many infections are not diagnosed or reported; additionally, we captured only cases of diarrhea diagnosed in the United States, and likely missed cases that resolved during travel. Because we did not adjust for under-reporting, our estimates of burden and risk may be more than 30 times lower than reality, and our estimates among travelers better reflect the subset of travelers who return to the United States with diarrhea, rather than the total number of travelers who acquire diarrhea while traveling [19, 20]. However, proportions of QNS isolates, associations between travel and QNS, and relative risk of QNS enteric infection by region should not be impacted by such under-reporting and can be used to guide decisions. Travelers in FoodNet sites may not be representative of U.S. travelers and the *Campylobacter* cases in this analysis may not be representative of cases in FoodNet; however, FoodNet data are population-based and may be more representative than data from studies based in travel clinics, which may be biased toward wealthier travelers or those more likely to seek care for travel-associated illnesses [18]. Furthermore, information on duration of travel is not collected in FoodNet, which could have increased the likelihood of travelers acquiring diarrhea. We defined patients as having a travel-associated infection if they traveled in the 7 days before illness onset; however, some of these infections may not have been acquired abroad or the patient’s infection may have resolved before returning to the US. Passenger data were available only for aviation travel since 2010 and not all data points represent unique travelers. Furthermore, because of limited data in NARMS, we were not able to assess the burden and risk of resistance to another drug, azithromycin that is commonly used to treat travelers’ diarrhea. Lastly, we were unable to link 8% of isolates in NARMS to FoodNet and 25% of isolates were from patients with no travel history information.

International travelers are at increased risk of acquiring QNS enteric bacterial infections and importing them into the United States, which can lead to onward transmission and domestic outbreaks of infections that are difficult to treat [4, 16]. Empiric antimicrobial treatment of travelers’ diarrhea is rarely required, is more common when travelers carry antibiotics with them [21], and may exacerbate the problem of acquisition and importation of antibiotic-resistant infections [3, 22, 23]. Fluroquinolones can also precipitate serious adverse events, such as rupture of the Achilles tendon, fatal dysrhythmias, and *C*. *difficile* infection [3]. Healthcare providers should counsel prospective travelers about diarrhea prevention and using safe and effective non-antibiotic medications, such as loperamide or bismuth subsalicylate, for relief of mild or moderate travelers’ diarrhea [3, 24, 25]. They should consider the regional prevalence of antimicrobial resistance if prescribing antibiotics for self-treatment during travel, and be aware that while azithromycin may be considered for travelers who require antibiotic treatment for severe diarrhea, azithromycin resistance is increasing [3]. Travelers with diarrhea should avoid using antibiotics to self-treat mild-to-moderate travelers’ diarrhea to reduce the risk of acquiring a QNS infection [3, 22, 26]. Healthcare providers should use antimicrobial susceptibility testing to guide treatment of returned ill travelers and counsel them on hygiene and handwashing practices to reduce the spread of diarrheal pathogens and thus the resistance genes they carry [3, 16]. Enhanced surveillance for antimicrobial resistance among pathogens isolated from returning travelers is essential to estimate risks, monitor trends in antimicrobial resistance associated with international travel, and guide prevention efforts.
